# Antibacterial Activity of Honey Samples from Ukraine

**DOI:** 10.3390/vetsci7040181

**Published:** 2020-11-20

**Authors:** Giovanni Cilia, Filippo Fratini, Matilde Marchi, Simona Sagona, Barbara Turchi, Leonora Adamchuk, Antonio Felicioli, Miroslava Kačániová

**Affiliations:** 1 Department of Veterinary Sciences, University of Pisa, Viale delle Piagge 2, 20159 Pisa, Italy; giovanni.cilia@vet.unipi.it (G.C.); matildemarchi23@hotmail.it (M.M.); barbara.turchi@unipi.it (B.T.); antonio.felicioli@unipi.it (A.F.); 2 Department of Pharmacy, University of Pisa, via Bonanno 6, 56126 Pisa, Italy; simona.sagona@unipi.it; 3 Department of Horse-Breeding and Beekeeping, National University of Life and Environmental Sciences of Ukraine, Henerala Rodimtseva Str.19, 03041 Kyiv, Ukraine; leonora.adamchuk@gmail.com; 4 Department of Fruit Sciences, Viticulture and Enology, Slovak University of Agriculture in Nitra, Tr. A. Hlinku 2, 949 76 Nitra, Slovakia; kacaniova.miroslava@gmail.com; 5 Department of Bioenergetics, Food Analysis and Microbiology, Institute of Food Technology and Nutrition, University of Rzeszow, Cwiklinskiej 1, 35-601 Rzeszow, Poland

**Keywords:** antibacterial activity, honey, minimal inhibitory concentration, minimal bactericidal concentration, melissopalynological analysis, physic-chemicals analysis

## Abstract

The employment of natural substances such as beehive products with a preventive and therapeutic purpose has been a widespread custom since ancient times. In this investigation, the antibacterial activity of 41 honey samples from different Ukraine regions has been evaluated. For each honey, melissopalynological and physico-chemical analysis were performed in order to determine botanical origin, pH, glucose and fructose contents and free acidity. So, antibacterial activity against *Staphylococcus* *aureus* CCM 4223, *Listeria monocytogenes* ATCC 7644, *Salmonella enterica* serovar Typhimurium CCM 3807 and *Escherichia coli* ATCC 25922 was assessed through the determination of MIC (Minimum Inhibitory Concentration) and MBC (Minimum Bactericidal Concentration) values by the microdilutions method. The results show that the most susceptible bacterial strain was L. monocytogenes. Its growth was inhibited at a honey concentration ranging from 0.094 to 0.188 g/mL. The most resistant bacterial strain was *S. aureus*. As concerns MBC values, *L. monocytogenes* was the most susceptible bacteria, while *S. aureus* was the most resistant. *Helianthus* spp. honeys was the most effective against all tested bacterial strains, followed by *Robinia* spp. and multifloral honeys. Promising results for MIC tests have been found for *Brassica* spp.

## 1. Introduction

Honey is an ancient remedy for treatment of infected wounds, which was recently “rediscovered” by the scientific community [1]. The first written reference to honey, a Sumerian tablet writing, dating back to 2100–2000 BC, mentions the use of honey as a drug and an ointment. Aristotle (384–322 BC) referred to pale honey as being “good as a salve for sore eyes and wounds”. In most ancient cultures, honey was used for both nutritional and medical purposes. The belief that honey is a nutrient, a drug and an ointment still survives today, and thus an alternative medicine branch, has been developed in recent years, offering treatments against many diseases including bacterial infections based on honey and other bee products [1].

The antimicrobial activity of honey is highly complex and still not fully recognized. To date, it has been established that several honey components play a crucial role for its antimicrobial properties [2]. Furthermore, royal jelly, beeswax and bee venom highlighted antimicrobial activity against a wide range of bacteria [3,4,5,6]. The antimicrobial activity of honey is due to physicochemical properties, such as the high content of reducing sugars, high viscosity, high osmotic pressure, low pH, low water activity (AW), low protein content and the presence of hydrogen peroxide [7,8]. None of these factors, taken individually, seem to be enough to explain the antimicrobial activity of honey, even if hydrogen peroxide and the bee-derived peptides with antibacterial activity were considered the major factors responsible for honey’s antibacterial activity [9,10,11]. Indeed, antibacterial activity seems to be strictly connected to the botanical origin of the plant, highlighting that the plant-derived component, especially hydrogen peroxide and polyphenols, are strictly involved in the antibacterial action [9,10,11]. As for other natural antimicrobials, such as essential oils and hydrolates, the antimicrobial activity of honey seems to derive from many factors, partially characterized. An important role among these factors is played by osmolarity, which was the main reason for honey being used over time as a sweetener ingredient, and also in long-term food preparations, for example, in Mediterranean pastries [12]. In honey, 95–99% of the dry matter is made of sugars, particularly fructose and glucose. The amount of water present in the product is a critical factor for the stability and safety of the product. For this reason, this microorganism group represents the main limit for the extension of the shelf-life of honey, considering that the minimum AW for the development of osmophilic yeasts is 0.61–0.62 [13]. A great number of studies have shown that honey has a broad-spectrum antibacterial activity against Gram-positive and Gram-negative bacteria. Honey antibacterial activity against *S. aureus*, *E. coli*, and *Salmonella* sp. could vary on the basis of two honey sample features: floral origin diversity and countries of origin [14,15,16,17,18,19]. It has been shown that honeys with low levels of phenol and tocopherol have the highest antibacterial activity against clinical isolates of *S. aureus, Proteus mirabilis*, *E. coli*, and *Shigella dysenteriae* [20].

Given the importance in a study that honeys might exert antimicrobial activity against pathogenic bacteria in a relationship with the botanical origins, the aim of this investigation was to evaluate the antimicrobial activity of 41 honey samples from different Ukraine regions against two Gram-positive bacterial strains (*S. aureus* CCM 4223 and *L. monocytogenes* ATCC 7644) and two Gram-negative bacterial strains (*Sal. enterica* servovar Typhimurium CCM 3807 and *E. coli* ATCC 25922).

## 2. Materials and Methods 

### 2.1. Samples Collection 

Forty-one pure crude honey samples were obtained directly from beekeepers from different regions of Ukraine. All samples were collected by the Department of Horse-Breeding and Beekeeping in Kyiv. All honey samples were stored in sterile plastic flasks at room temperature in the dark.

In all honey samples, the absence of antibiotics is regulated by legislation (Ukraine SCBM Orden n. 143, 23/12/2004, Ukraine SCVM Order n. 40, 28/05/2003). Beekeepers demonstrated that the honey collected during the year of this investigation was tested to the presence of antibiotic residues in the honey. The analysis, carried out by external laboratories through HPLC, reported the absence of tetracyclines, sulphonamides and streptomycin in all honeys.

### 2.2. Melissopalynological Analysis and Physico-Chemical Analysis 

The botanical origin of the samples of honey was based on the pollen spectrum, thought melissopalynological analysis [21]. The microscopic analysis of each honey for the palynological components identification were carried out according to the melissopalynological method. Briefly, 10 g of honey was mixed with 50 mL of distilled water and was centrifuged for 10 min at 3000 rpm. Afterwards, the precipitate was suspended again in distilled water and centrifuged for 7 min at 3000 rpm. The final precipitate was transferred in a microscope slide, dried and fixed using glycerin jelly for permanent preparations. Pollen grain identification was performed by optical microscope with total magnification (400× and 1000×) using as a reference a pollen collection from the Department of Horse-Breeding and Beekeeping in Kyiv and different pollen morphology guides. Only in order to characterize the tested honey samples, physico-chemical parameters were analyzed according the Official Methods of Analysis of Association of Official Analytical Chemists [22] and the Harmonised Methods of the European Honey Commission [23]. All honey samples were analyzed during the same time period to uniform the conditions and then comparability.

To pH measurement, 5 g of honey samples was diluted with 20 mL of distilled water and mixed thoroughly [22]. The pH was measured using the Digital pH Meter (pH-526 WTW Germany). 

Free acidity was determined dissolving 10 g of honey samples in 75 mL of CO2-free water [22]. The electrode of the pH meter was immersed in the solution, stirred with a magnetic stirrer, and titrated to pH 8.5 by adding 0.05 N NaOH solution.

Glucose and fructose content were identified using a High-Pressure Liquid Chromatograph (HPLC), composed by Alliance Separations Module (Waters), Aminex HPX-87H 300 × 7.8 mm column, Cation-H precolumn and Refractive Index Detector (Waters). To measure sugars, 0.4 g of honey sample was dissolved in 3 m of milli-Q water and transferred volumetric flask. The solution was well mixed, and it was filtered through a 0.2 μm Millipore filter and an additional 10× dilution was made with HPLC eluent. 

### 2.3. Bacterial Strains 

Four bacterial strains obtained from international type culture collections were tested: *S. aureus* CCM 4223, *L. monocytogenes* ATCC 7644, *Sal. enterica* serovar. Typhimurium CCM 3807 and *E. coli* ATTC 25922.

Strains were stored at −80 °C in a glycerol suspension until their use. Before the antibacterial activity determinations, the strains were cultured in BHI (Brain Hearth Infusion, Oxoid, Milan, Italy) broth for 24 h at 37 °C in aerobic conditions.

### 2.4. Minimal Inhibitory Concentration (MIC) and Minimal Bactericidal Concentration (MBC)

Honey samples were weighed (1.5 g) and diluted in 2 mL of BHI broth. In order to determine the MIC values, a microdilutions method was employed, using 96-well polypropylene microtiter plates. In the first empty row of wells, 190 μL of stock solution with honey and BHI was distributed, then twofold dilutions were performed. A total of 10 µL of bacterial suspension was added to each well to reach a final volume of 200 µL [24]. 

Bacterial suspensions were adjusted using the McFarland standard turbidity scale in order to obtain approximately 1.5 × 10^8^ cfu/mL for each strain. As a negative control, sterile BHI broth was used. Microplates were incubated at 37 °C for 24 h in a humid chamber in aerobic conditions.

MBC was determined by streaking one drop from each well that showed a concentration of honey equal to or higher than the MIC value on TSA (Trypticase Soy Agar) to evaluate microbial growth. TSA plates were incubated at 37 °C for 24 h. MBC values were determined as the lowest concentrations that did not allow colonies to grow.

### 2.5. Statistical Analysis

The MIC and MBC results were expressed as mode (the value that appears most often) and the comparison of the antibacterial activity of the samples was evaluated by applying *t*-test. *p* ≤ 0.05 values were considered to indicate statistically significant differences.

## 3. Results

### 3.1. Melissopalynological and Physico-Chemical Analysis

Honey botanical origins through melissopalynological analysis and pH, glucose, fructose and free acidity are reported in Table 1 in relation with the provenience region. The sampled honeys were divided as follows: 11 samples of *Helianthus* honey, 10 of *Robinia* honey, 3 of *Brassica* honey, 2 of *Tilia* honey, 1 of *Coriandrum sativum* honey, 1 of *Echium vulgare* honey, 8 of multiflower honey, 3 of organic multiflower honey and 2 of multiflower honey from a medicinal plant.

Moreover, in accordance with the average values characteristic for each monofloral honey, the parameter of pH, glucose, fructose and free acidity, evaluated in this investigation, are in accordance with the botanical origins of honey [22,23,25].

### 3.2. Minimal Inhibitory Concentration and Minimal Bactericidal Concentration)

The MIC and MBC values, reported in Table 2, showed that the most susceptible bacterial strain against the tested honey was *L. monocytogenes*, while *E. coli* and *Sal. enterica* were less susceptible. *S. aureus* was the most resistant bacterium. *E. coli* was the most susceptible to *Helianthus* spp. (samples 8, 13, 16, 39 and 40), *Robinia* spp. (25 and 33), and multifloral (4, 7, 11, 12 and 35) honeys, which inhibited its growth at a concentration of 0.188 g/mL. *Helianthus* spp. honey (sample 14) is the only monofloral tested honey that inhibited *Sal. enterica* growth at a concentration of 0.094 g/mL. All the remaining honey samples inhibited *Sal. enterica* growth at a concentration of 0.188 g/mL or 0.375 g/mL. The most resistant bacterial strain, *S. aureus*, has been inhibited only by two out of eleven *Helianthus* spp. honeys (samples 13 and 16) at a concentration of 0.094 g/mL and by one out of three *Brassica* spp. honeys (sample 6) and one out of ten *Robinia* spp. honeys (sample 17) at a concentration of 0.188 g/mL. The other tested honeys inhibited its growth at a concentration of 0.375 g/mL. *L. monocytogenes* was the most susceptible bacterial strain to all tested honeys. All tested honeys inhibited *L. monocytogenes* growth at a concentration ranging from 0.094 to 0.375 g/mL.

*Helianthus* spp. honeys was the most effective against all tested bacterial strains, followed by *Robinia* spp. and multifloral honeys (*p* = 0.01). Only *Sal. enterica* and *L. monocytogenes* were inhibited at a concentration of 0.188 g/mL by a wide range of tested monofloral and multifloral honeys, including *E. vulgare* and *C. sativum*. The most effective honey against all tested bacterial strains was *Helianthus* spp. honey (*p* = 0.02), followed by multifloral honeys, while the activity of *Robinia* spp. was moderate. Monofloral and multifloral honeys showed no differences among their antibacterial activity (*p* = 0.17). Thus, the antibacterial activity of multifloral honeys could be due to the same components present in monofloral honeys.

## 4. Discussion

The antibacterial activity of honey is strictly related to botanical source, the metabolism of honey bees, environmental-seasonal-climatic processing procedures and storage conditions, which have a strong influence on the physical and chemical property of this “medical” food, including antimicrobial properties [14,15,16,17,26,27,28,29,30]. Antimicrobial compounds of honey can be divided in two different groups: those with peroxide action and those with non-peroxide action. Hydrogen peroxide, especially derived from plants, is the component responsible for the antibacterial activity of honey compound with peroxide action [26,31,32]. It is produced under aerobic conditions by glucose oxidase from glucose [33]. Other factors are involved in the antimicrobial and antibacterial activity of honey: high osmolarity, acidity, antioxidant activity, nitric-oxide, limphocytic and phagocytic activity increase, and prostaglandins’ reduction. These factors can inhibit bacterial growth and toxin production and affect bacterial biofilm expression, and bacterial cell wall structure [34].

The great antibacterial activity of *Helianthus* spp. honey could be related to the high value (compared to other honeys) of acidity (mean value 25.5). Free acidity has affected against bacteria in synergy with the pH action and sugars amount [29,35,36,37]. Fructose and sucrose, reported in high levels in *Robinia* spp. monofloral honey (mean value 43.2), could be involved in antibacterial activity. The high level of glucose (mean value 50.3) reported for *Brassica* honey and low level of glucose reported for *Robinia* spp. honey (mean value 26.5) indicate that this sugar could not play a role in antibacterial activity, or its activity is exclusively against specific bacteria, in this investigation *S. aureus* [37,38]. The antibacterial activity of honey was deeply investigated, both for monofloral and multifloral samples. According to these data, several bacteria are inhibited by honey, and in some cases are killed. Moreover, all tested honey evidenced higher values of MBC in comparison to MIC ones. These differences, usually common for many other antimicrobial substances derived from natural product [6,24,39] have been also reported in other several researches on honey [11,40,41,42]. *L. monocytogenes* was one of the most susceptible among tested bacteria in accordance with other data available in literature [43,44,45]. Its growth was inhibited by the monofloral honey of *Helianthus* spp. [26,43,44,45] and *Trifolium spp.* [26] and by different multifloral honeys [43,44,45]. *Robinia* spp. and *Tilia spp.* monofloral honeys were less effective against *L. monocytogenes* [43,44,45]. The high susceptibility of *L. monocytogenes* to *Robinia* spp. and *Tilia spp.* monofloral honeys here reported seems to be related to the different strains employed or tests performed to evaluate MIC. In this investigation, the antibacterial activity of honeys against *L. monocytogenes* ATCC 7644 was assessed by the determination of MIC in a microdilution method, while, in the other studies, the agar-well diffusion method on *L. monocytogenes* ATCC 19115 was performed [43,44,45].

In this investigation, in accordance with other studies [43,44,45], *Helianthus* spp. honeys showed the highest antibacterial activity against *E. coli*, followed by *Tilia* and *Robinia* spp. Different honeys, in general, showed inhibitory activity against *E. coli* growth [26,41,46,47,48,49], but other authors found that multifloral honeys tested against *E. coli* showed a lack of significant susceptibility [50,51]. *Robinia* spp. honeys showed a good antibacterial activity against *E. coli* in accordance with Chang et al. (2011), but the *Tilia* honeys tested show a low activity. In the present investigation, detected MIC values concerning *Tilia* honey against *E. coli* are, instead, in accordance with Fidaleo et al. (2011) and Vica et al. (2014). It is possible to assume that the differences in the results can be related to the different bacterial strains employed. Indeed, *E. coli* ATCC 25922 used in this investigation is the same strain employed in two studies [45,52].

In this investigation, *Sal. Enterica* showed a wide spectrum of response with tested honeys, in accordance with other studies [26,47,53]. Monofloral *Helianthus* spp. honey inhibited different pathogens, among which is *Sal. enterica* [26]. Chestnut honeys and multifloral honeys from Sicily were tested against *Sal. enterica* serovar Infantis evidencing great antimicrobial activity [54].

In this investigation, the most resistant bacterial strain was *S. aureus*, for which the higher MIC and MBC values for all tested honeys have been reported, even if this bacterium is usually described as the most sensitive to honey’s antimicrobial activity. Monofloral and multifloral honeys are able to inhibit *S. aureus* growth [7,44,45,52,55]. *S. aureus* was the most sensitive bacterial strain to *Helianthus* spp. and *Tilia* spp. monofloral honeys. Moreover, in literature, *Robinia* spp. and *Brassica napus* monofloral honeys, showed antibacterial activity against *S. aureus* [45,56,57]. On the other hand, several studies highlighted the high susceptibility of *S. aureus*, especially compared to Gram-negative bacteria [9,11,34,58,59]. These differences in the antibacterial activity could be related to the geographical provenience of honey, the environment of collection zone, the genetic of honeybee and its family could influence the properties of honey.

## 5. Conclusions

In conclusion, the different antibacterial activity of honey against the same bacterial strains tested in this investigation could be attributed to several factors, including provenience and physico-chemical properties (such as pH, glucose and fructose content and free acidity). It could be possible that the different bacterial strains employed resulted in a different susceptibility degree within different antimicrobial activity evaluation tests. Moreover, the antibacterial activity could be related to different *Robiania* spp., *Helianthus* spp., *Brassica* spp. and *Tilia* spp. monofloral honeys’ proveniences. The geographical provenience of honey, the surrounding environment of the collection zone, and the genetics of the honeybee and its family could influence the properties of honey and its antibacterial activity [60,61,62,63].

Moreover, the added antimicrobial peptides (AMPs) or antibacterial peptides, in different concentrations for each honeybee family, could play a role in the different antibacterial activity of honey. The physical-chemical features derived from plant compounds and honeybee factors change in each honey, as well as in honey derived from the same botanical source.

## Figures and Tables

**Table 1:** Characteristic of honey samples tested.

Sample	Botanical Origin	Melissopalynology	Provenience	Physico-Chemical Analyses			
				pH	Glucose ^1^	Fructose ^1^	Acidity ^2^
1	*Brassica* spp.	*Brassica* 99% *Taraxacum* 1%	Zhytomyr region	4.2	47.5	42.3	14.8
2	Multifloral (organic honey)	*Medicago* 14% *Helianthus* 12% *Phacelia* 11% *Tilia* 8%	Ivano-Frankivsk region	3.8	27.9	39.9	19.7
3	Multifloral (medicinal plants)	*Eucalyptus* 21% *Phacelia* 12% *Lavandula* 8% *Thymus* 5%	Kiev region	3.9	25.5	37.4	30.4
4	Multifloral	*Castanea* 15% *Tilia* 11% *Thymus* 8% *Phacelia* 2%	Zhytomyr region	3.8	28.9	42.5	33.0
5	*Echium vulgare*	*Echium* 61% *Robinia* 12% *Phacelia* 12% *Thymus* 9%	Kherson region	3.8	30.1	41.9	28.0
6	*Brassica* spp.	*Brassica* 97% *Taraxacum* 3%	Zhytomyr region	3.8	47.1	44.7	10.7
7	Multifloral (organic honey)	*Eucalyptus* 18% *Phacelia* 7% *Thymus* 3%	Ivano-Frankivsk region	3.9	29.0	37.3	22.2
8	*Helianthus* spp.	*Helianthus* 69% *Trifolium* 12% *Tilia* 10% *Medicago* 9%	Kharkiv region	3.8	37.0	42.8	24.7
9	Multifloral	*Rosmarinus* 20% *Taraxacum* 10% *Brassica* 8%	Kiev region	3.7	29.4	37.7	23.9
10	*Robinia* spp.	*Robinia* 25% *Phacelia* 12% *Taraxacum* 3%	Ukraine, industrial honey	3.9	26.1	41.3	11.3
11	Multifloral	*Eucalyptus* 21% *Castanea* 13% *Phacelia* 11% *Trifolium* 5%	Kiev region	3.8	27.0	37.6	27.1
12	Multifloral (organic honey)	*Castanea* 17% *Aesculus* 12% *Phacelia* 11% *Trifolium* 8% *Thymus* 5%	Ivano-Frankivsk region	3.9	27.0	37.5	16.0
13	*Helianthus* spp.	*Helianthus* 71% *Trifolium* 11% *Medicago* 8%	Zhytomyr region	3.9	37.5	43.9	28.6
14	*Robinia* spp.	*Robinia* 31% *Castanea* 11% *Taraxacum* 4%	Ukraine, industrial honey	3.8	26.4	43.2	11.3
15	*Robinia* spp.	*Robinia* 27% *Phacelia* 10% *Castanea* 4%	Kiev region	3.5	27.1	42.4	11.6
16	*Helianthus* spp.	*Helianthus* 68% *Medicago* 18% *Trifolium* 14%	Zhytomyr region	3.9	36.9	43.4	21.0
17	*Robinia* spp.	*Robinia* 29% *Phacelia* 11% *Taraxacum* 6%	Ukraine, industrial honey	4.0	28.2	41.2	11.5
18	*Helianthus* spp.	*Helianthus* 80% *Medicago* 11% *Trifolium* 9%	Kharkiv region	3.9	37.0	42.5	27.9
19	Multifloral	*Rosmarinus* 17% *Taraxacum* 12% *Brassica* 7%	Zhytomyr region	3.8	32.5	40.7	34.6
20	*Helianthus* spp.	*Helianthus* 72% *Medicago* 12% *Trifolium* 10% *Phacelia* 6%	Kharkiv region	3.8	37.5	44.6	28.6
21	*Robinia* spp.	*Robinia* 35% *Phacelia* 9% *Taraxacum* 6%	Donetsk region	3.8	26.4	43.2	11.3
22	Multifloral	*Eucalyptus* 18% *Phacelia* 13% *Castanea* 9% *Thymus* 3%	Donetsk region	3.8	31.4	37.4	18.1
23	*Helianthus* spp.	*Helianthus* 90% *Medicago* 7% *Trifolium* 3%	Donetsk region	3.9	37.6	43.1	24.2
24	*Brassica* spp.	*Brassica* 98% *Taraxacum* 2%	Kiev region	4.1	49.3	42.4	13.3
25	*Robinia* spp.	*Robinia* 21% *Phacelia* 10% *Castanea* 8%	Ukraine, industrial honey	3.9	25.6	45.0	10.8
26	*Robinia* spp.	*Robinia* 34% *Castanea* 13% *Phacelia* 11% *Taraxacum* 5%	Ukraine, industrial honey	4.0	26.9	44.1	11.3
27	*Robinia* spp.	*Robinia* 25% *Castanea* 9% *Taraxacum* 2%	Ukraine, industrial honey	4.0	26.2	43.5	10.9
28	Multifloral	*Castanea* 17% *Aesculus* 12% *Trifolium* 11% *Thymus* 8% *Lavandula* 5%	Zhytomyr region	3.9	31.9	37.9	20.8
29	*Tilia* spp.	*Tilia* 25% *Castanea* 12%	Kiev region	4.5	28.5	41.6	24.3
30	*Tilia* spp.	*Tilia* 31% *Castanea* 14%	Kiev region	4.2	30.1	41.9	19.8
31	Multifloral (medicinal plants)	*Eucalyptus* 23% *Lavandula* 13% *Phacelia* 9% *Trifolium* 4%	Kiev region	3.9	31.8	38.7	26.2
32	*Helianthus* spp.	*Helianthus* 75% *Medicago* 13% *Trifolium* 12%	Zhytomyr region	3.8	38.2	44.8	20.3
33	*Robinia* spp.	*Robinia* spp. 39% *Castanea* 12% *Phacelia* 11%	Ukraine, industrial honey	3.7	26.0	45.7	10.1
34	*Coriandrum sativum*	*Coriandrum* 99% *Castanea* 1%	Ukraine, industrial honey	3.9	35.7	35.9	30.0
35	Multifloral	*Taraxacum* 18% *Brassica* 14% *Rosmarinus* 3% *Tilia* 2%	Zhytomyr region	3.9	25.3	39.4	18.2
36	*Robinia* spp.	*Robinia* spp. 28% *Phacelia* 12% *Castanea* 7% *Taraxacum* 2%	Ukraine, industrial honey	4.0	26.2	42.5	8.7
37	Multifloral	*Eucalyptus* 17% *Castanea* 16% *Trifolium* 11% *Thymus* 7%	Zhytomyr region	3.9	30.9	37.2	19.8
38	*Helianthus* spp.	*Helianthus* 81% *Trifolium* 9% *Medicago* 6%	Zhytomyr region	3.8	37.0	44.9	30.3
39	*Helianthus* spp.	*Helianthus* 99% *Trifolium* 1%	Zhytomyr region	3.8	36.8	45.0	27.7
40	*Helianthus* spp.	*Helianthus* 91% *Medicago* 9%	Zhytomyr region	3.9	36.9	43.8	24.6
41	*Helianthus* spp.	*Helianthus* 94% *Trifolium* 6%	Kharkiv region	3.8	36.6	43.5	22.1

Note. ^1^: expressed in g/100 g of honey; ^2^: expressed in meq/kg of honey.

**Table 2:** Results of Minimum Inhibitory Concentration (MIC) and Minimum Bactericidal Concentration (MBC) tests of honey (modal values).

Honey Botanical Origin	Sample	Bacterial Strain	MIC Mode	MBC Mode
*Brassica* spp.	1	*E. coli*	0.375 g/mL	>0.750 g/mL
		*Salmonella*	0.188 g/mL	>0.750 g/mL
		*L. monocytogenes*	0.188 g/mL	0.750 g/mL
		*S. aureus*	0.375 g/mL	>0.750 g/mL
	6	*E. coli*	0.375 g/mL	0.750 g/mL
		*Salmonella*	0.375 g/mL	>0.750 g/mL
		*L. monocytogenes*	0.188 g/mL	>0.750 g/mL
		*S. aureus*	0.188 g/mL	>0.750 g/mL
	24	*E. coli*	0.375 g/mL	0.375 g/mL
		*Salmonella*	0.188 g/mL	0.375 g/mL
		*L. monocytogenes*	0.188 g/mL	0.375 g/mL
		*S. aureus*	0.375 g/mL	>0.750 g/mL
Organic Multifloral	2	*E. coli*	0.375 g/mL	0.375 g/mL
		*Salmonella*	0.375 g/mL	0.750 g/mL
		*L. monocytogenes*	0.188 g/mL	0.188 g/mL
		*S. aureus*	0.375 g/mL	0.750 g/mL
	7	*E. coli*	0.188 g/mL	0.750 g/mL
		*Salmonella*	0.188 g/mL	0.750 g/mL
		*L. monocytogenes*	0.094 g/mL	0.188 g/mL
		*S. aureus*	0.375 g/mL	0.375 g/mL
	12	*E. coli*	0.188 g/mL	0.750 g/mL
		*Salmonella*	0.375 g/mL	0.375 g/mL
		*L. monocytogenes*	0.094 g/mL	0.188 g/mL
		*S. aureus*	0.375 g/mL	0.375 g/mL
Medicinal Plant Multifloral	3	*E. coli*	0.375 g/mL	>0.750 g/mL
		*Salmonella*	0.375 g/mL	>0.750 g/mL
		*L. monocytogenes*	0.375 g/mL	0.750 g/mL
		*S. aureus*	0.375 g/mL	>0.750 g/mL
	31	*E. coli*	0.375 g/mL	0.750 g/mL
		*Salmonella*	0.375 g/mL	0.750 g/mL
		*L. monocytogenes*	0.375 g/mL	0.750 g/mL
		*S. aureus*	0.375 g/mL	0.750 g/mL
Multifloral	4	*E. coli*	0.188 g/mL	0.188 g/mL
		*Salmonella*	0.188 g/mL	0.375 g/mL
		*L. monocytogenes*	0.375 g/mL	0.750 g/mL
		*S. aureus*	0.375 g/mL	0.750 g/mL
	9	*E. coli*	0.375 g/mL	0.375 g/mL
		*Salmonella*	0.188 g/mL	0.375 g/mL
		*L. monocytogenes*	0.188 g/mL	0.188 g/mL
		*S. aureus*	0.375 g/mL	0.375 g/mL
	11	*E. coli*	0.188 g/mL	0.375 g/mL
		*Salmonella*	0.188 g/mL	0.375 g/mL
		*L. monocytogenes*	0.188 g/mL	0.188 g/mL
		*S. aureus*	0.375 g/mL	>0.750 g/mL
	19	*E. coli*	0.375 g/mL	0.750 g/mL
		*Salmonella*	0.375 g/mL	0.750 g/mL
		*L. monocytogenes*	0.188 g/mL	0.750 g/mL
		*S. aureus*	0.375 g/mL	0.375 g/mL
	22	*E. coli*	0.375 g/mL	0.375 g/mL
		*Salmonella*	0.375 g/mL	0.750 g/mL
		*L. monocytogenes*	0.188 g/mL	0.375 g/mL
		*S. aureus*	0.375 g/mL	0.750 g/mL
	28	*E. coli*	0.375 g/mL	0.375 g/mL
		*Salmonella*	0.375 g/mL	0.375 g/mL
		*L. monocytogenes*	0.188 g/mL	0.188 g/mL
		*S. aureus*	0.375 g/mL	0.375 g/mL
	35	*E. coli*	0.188 g/mL	0.750 g/mL
		*Salmonella*	0.188 g/mL	0.750 g/mL
		*L. monocytogenes*	0.188 g/mL	0.188 g/mL
		*S. aureus*	0.375 g/mL	0.750 g/mL
	37	*E. coli*	0.375 g/mL	>0.750 g/mL
		*Salmonella*	0.375 g/mL	0.375 g/mL
		*L. monocytogenes*	0.188 g/mL	0.750 g/mL
		*S. aureus*	0.375 g/mL	0.750 g/mL
*E. vulgare*	5	*E. coli*	0.375 g/mL	0.750 g/mL
		*Salmonella*	0.188 g/mL	0.375 g/mL
		*L. monocytogenes*	0.188 g/mL	0.375 g/mL
		*S. aureus*	0.375 g/mL	0.750 g/mL
*Helianthus* spp.	8	*E. coli*	0.188 g/mL	0.750 g/mL
		*Salmonella*	0.188 g/mL	>0.75 g/mL
		*L. monocytogenes*	0.375 g/mL	0.750 g/mL
		*S. aureus*	0.375 g/mL	>0.750 g/mL
	13	*E. coli*	0.188 g/mL	0.375 g/mL
		*Salmonella*	0.094 g/mL	0.375 g/mL
		*L. monocytogenes*	0.094 g/mL	0.188 g/mL
		*S. aureus*	0.094 g/mL	0.188 g/mL
	16	*E. coli*	0.188 g/mL	0.188 g/mL
		*Salmonella*	0.188 g/mL	0.188 g/mL
		*L. monocytogenes*	0.094 g/mL	0.188 g/mL
		*S. aureus*	0.094 g/mL	0.375 g/mL
	18	*E. coli*	0.375 g/mL	0.750 g/mL
		*Salmonella*	0.375 g/mL	>0.750 g/mL
		*L. monocytogenes*	0.188 g/mL	0.750 g/mL
		*S. aureus*	0.375 g/mL	0.750 g/mL
	20	*E. coli*	0.375 g/mL	0.750 g/mL
		*Salmonella*	0.375 g/mL	>0.750 g/mL
		*L. monocytogenes*	0.375 g/mL	0.750 g/mL
		*S. aureus*	0.375 g/mL	>0.750 g/mL
	23	*E. coli*	0.375 g/mL	0.375 g/mL
		*Salmonella*	0.188 g/mL	0.375 g/mL
		*L. monocytogenes*	0.188 g/mL	0.188 g/mL
		*S. aureus*	0.375 g/mL	0.375 g/mL
	32	*E. coli*	0.375 g/mL	0.750 g/mL
		*Salmonella*	0.375 g/mL	0.750 g/mL
		*L. monocytogenes*	0.188 g/mL	0.188 g/mL
		*S. aureus*	0.375 g/mL	0.750 g/mL
	38	*E. coli*	0.375 g/mL	0.750 g/mL
		*Salmonella*	0.375 g/mL	>0.750 g/mL
		*L. monocytogenes*	0.188 g/mL	0.188 g/mL
		*S. aureus*	0.375 g/mL	0.750 g/mL
	39	*E. coli*	0.188 g/mL	>0.75 g/mL
		*Salmonella*	0.188 g/mL	0.75 g/mL
		*L. monocytogenes*	0.188 g/mL	0.188 g/mL
		*S. aureus*	0.375 g/mL	0.375 g/mL
	40	*E. coli*	0.188 g/mL	0.188 g/mL
		*Salmonella*	0.375 g/mL	0.75 g/mL
		*L. monocytogenes*	0.094 g/mL	0.188 g/mL
		*S. aureus*	0.375 g/mL	0.75 g/mL
	41	*E. coli*	0.375 g/mL	0.75 g/mL
		*Salmonella*	0.375 g/mL	>0.75 g/mL
		*L. monocytogenes*	0.375 g/mL	0.375 g/mL
		*S. aureus*	0.375 g/mL	0.75 g/mL
*Robinia* spp.	10	*E. coli*	0.375 g/mL	0.750 g/mL
		*Salmonella*	0.375 g/mL	0.750 g/mL
		*L. monocytogenes*	0.375 g/mL	0.750 g/mL
		*S. aureus*	0.375 g/mL	>0.750 g/mL
	14	*E. coli*	0.375 g/mL	>0.750 g/mL
		*Salmonella*	0.375 g/mL	>0.750 g/mL
		*L. monocytogenes*	0.375 g/mL	0.750 g/mL
		*S. aureus*	0.375 g/mL	0.750 g/mL
	15	*E. coli*	0.375 g/mL	0.750 g/mL
		*Salmonella*	0.375 g/mL	>0.750 g/mL
		*L. monocytogenes*	0.375 g/mL	0.750 g/mL
		*S. aureus*	0.375 g/mL	0.750 g/mL
	17	*E. coli*	0.375 g/mL	0.750 g/mL
		*Salmonella*	0.375 g/mL	0.750 g/mL
		*L. monocytogenes*	0.188 g/mL	0.375 g/mL
		*S. aureus*	0.188 g/mL	>0.750 g/mL
	21	*E. coli*	0.375 g/mL	0.750 g/mL
		*Salmonella*	0.375 g/mL	>0.750 g/mL
		*L. monocytogenes*	0.375 g/mL	0.750 g/mL
		*S. aureus*	0.375 g/mL	>0.750 g/mL
	25	*E. coli*	0.188 g/mL	0.750 g/mL
		*Salmonella*	0.188 g/mL	0.750 g/mL
		*L. monocytogenes*	0.188 g/mL	0.750 g/mL
		*S. aureus*	0.375 g/mL	>0.750 g/mL
	26	*E. coli*	0.375 g/mL	0.750 g/mL
		*Salmonella*	0.375 g/mL	>0.750 g/mL
		*L. monocytogenes*	0.375 g/mL	0.750 g/mL
		*S. aureus*	0.375 g/mL	>0.750 g/mL
	27	*E. coli*	0.375 g/mL	>0.750 g/mL
		*Salmonella*	0.188 g/mL	>0.750 g/mL
		*L. monocytogenes*	0.188 g/mL	0.750 g/mL
		*S. aureus*	0.375 g/mL	>0.750 g/mL
	33	*E. coli*	0.188 g/mL	0.750 g/mL
		*Salmonella*	0.188 g/mL	>0.750 g/mL
		*L. monocytogenes*	0.188 g/mL	0.750 g/mL
		*S. aureus*	0.375 g/mL	0.375 g/mL
	36	*E. coli*	>0.750 g/mL	>0.750 g/mL
		*Salmonella*	>0.750 g/mL	>0.750 g/mL
		*L. monocytogenes*	0.188 g/mL	0.750 g/mL
		*S. aureus*	0.375 g/mL	0.750 g/mL
*Tilia* spp.	29	*E. coli*	0.375 g/mL	0.375 g/mL
		*Salmonella*	0.188 g/mL	0.375 g/mL
		*L. monocytogenes*	0.188 g/mL	0.375 g/mL
		*S. aureus*	0.375 g/mL	>0.750 g/mL
	30	*E. coli*	0.375 g/mL	>0.750 g/mL
		*Salmonella*	0.188 g/mL	0.750 g/mL
		*L. monocytogenes*	0.375 g/mL	>0.750 g/mL
		*S. aureus*	0.375 g/mL	0.750 g/mL
*C. sativum*	34	*E. coli*	0.375 g/mL	>0.750 g/mL
		*Salmonella*	0.188 g/mL	0.750 g/mL
		*L. monocytogenes*	0.375 g/mL	0.750 g/mL
		*S. aureus*	0.375 g/mL	>0.750 g/mL
